# Thematic series on supramolecular chemistry

**DOI:** 10.3762/bjoc.5.76

**Published:** 2009-12-11

**Authors:** Christoph A Schalley

**Affiliations:** 1 Institut für Chemie und Biochemie der Freien Universität Berlin, Takustr. 3, D-14195 Berlin, Germany

*“Some might say that supramolecular systems rescued physical organic chemistry. The discovery of crown ethers gave the field new recognition: molecular recognition.” *[1]

As the above citation from a paper by Julius Rebek and his coworkers indicates, supramolecular chemistry at its beginning gave new impetus to physical organic chemistry, which at that time had got trapped in ever more detailed kinetic studies. Early on, the nature of non-covalent interactions was of great interest. The first synthetic host-guest complexes were studied with respect to their components’ ability to bind selectively to each other through weak interactions. Mostly cations were used as the guests, because they provided rather strong binding interactions due to their charge and formed quite directional bonds.

Since these first steps supramolecular chemistry has matured into a research field in its own right. A large number of concepts have been developed which increased the binding strengths due to preorganization and the chelate effect. These concepts have been successfully transferred to neutral and anionic hosts. Nowadays, multivalent interactions start to play a significant role for host-guest chemistry.

But supramolecular chemistry is much more than molecular recognition. Concepts such as templated synthesis, (hierarchical) self-assembly, and self-sorting have made supramolecular synthesis a powerful tool to construct large and complex chemical architecture from simple building blocks with an inherent program of well-designed binding sites. Based on these concepts, functional supramolecules were developed, among them molecular switches, logic gates, molecular containers, elevators, valves and springs, supramolecular catalysts and many more.

Fixing such functional supramolecules in a suitable way, for example on nanoparticles, at interfaces or in membranes can be a way to generate even novel materials with interesting macroscopic effects.

This exciting development has been accompanied by a development of new methods able to monitor the sometimes quite fast dynamics of supramolecular systems. With this, supramolecular chemistry has become fruitful also for other areas in chemistry.

It is a great pleasure to act as the editor of a thematic series on supramolecular chemistry and I would like to thank very much all contributors to this thematic series for their excellent contributions.

Christoph A. Schalley

Berlin, November 2009
